# Genetic modification and yield risk: A stochastic dominance analysis of corn in the USA

**DOI:** 10.1371/journal.pone.0222156

**Published:** 2019-10-10

**Authors:** Elizabeth Nolan, Paulo Santos

**Affiliations:** 1 Affiliation School of Economics, The University of Sydney, Sydney, NSW, Australia; 2 Affiliation Dept Economics, Monash University, Caulfield, Vic, Australia; Pennsylvania State University, UNITED STATES

## Abstract

Production risk has been ignored in most of the analysis of GM technology, which has mostly focused on its effects on mean yield. We use stochastic dominance to quantify the effect of GM traits on the entire distribution of yields in corn in the USA under a wide range of growing conditions. Although no GM hybrid outperforms conventional hybrids under all growing conditions, we present evidence that most GM hybrids can be considered as improvements of the yield distribution.

## Introduction

Production risk is an essential feature of agricultural production. In the absence of full insurance, risk averse producers will decide on input use taking into account the fact that the yield distribution as a whole (and not just mean yield) can be influenced by input use [1–3]. As a result, production risk may partially determine the adoption of new technologies [4].

In this article we quantify the changes in yield distribution associated with the development of genetically modified (GM) corn. The presence of the genes associated with the production of a protein found in the soil bacterium *Bacillus thuringiensis* which is toxic to lepidopterous insects allows for an almost complete protection against the most economically important pests of corn in the USA, the European corn borer and corn rootworm [5, 6]. The clearly superior level of control of these two pests when using GM traits, when compared with the use of pesticides, certainly helps explain their rapid adoption: by 2014, less than twenty years after they were first released, GM hybrids represented around 93% of the corn acreage in the United States [7], and have remained at that level since then.

Most of the earlier work that quantifies the economic impacts of GM crops (including corn) focused on the effects on mean yield [8–10], even though the complete evaluation of the impact of new technologies requires explicit consideration of changes in production risk [11]. The limited literature on the effect of GM traits on yield variability is based on the estimation of stochastic production functions, pioneered by [12] and further developed by [13]. Existing analysis [14, 15] suggests that the presence of GM traits lowers the cost of risk, but the effects of GM traits on risk are possibly heterogeneous with respect to the specific GM trait under consideration [14] or agroecological conditions [15].

We contribute to this discussion in two ways. Firstly, we analyze a large dataset of experimental trials of corn hybrids run in the ten most important corn-producing states in the United States, over the period 1997-2009 (ie, the first 13 years since the commercial introduction of GM hybrids). Because our data extends beyond the Corn Belt, an area characterized by low level of yield risk for corn [16], we are able to measure the effect of GM traits on the entire yield distribution where yield variability likely matters most. Hence we overcome one limitation of earlier analysis, its limited geographical coverage.

Secondly, the use of parametric approaches such as stochastic production functions to estimate the effect of GM traits on the different moments of the yield distribution, makes a conclusive judgement about the desirability of the overall effect of this technology dependent on specific assumptions about the decision criterion of producers (namely, functional form of the utility function, magnitude of risk aversion, etc.)—see [14] for an example of this approach. An alternative approach is the use stochastic dominance, a technique that allows us to rank the desirability of the conditional yield distributions generated by different technologies (here, GM hybrids versus conventional hybrids) under minimal assumptions both regarding decision makers’ objectives [17] and the relation between input use and the moments of the yield distribution.

The rest of this article proceeds as follows. In the next section we present an intuitive explanation of the approach we use, stochastic dominance, and how it allows us to rank different alternatives. We then proceed to present the data and the results of our analysis. We conclude with a discussion of what these results may mean for adoption at farmer level, as well as a few unexplored questions.

## Estimating the effect of input use on production risk: Stochastic dominance

In this section we present an intuitive explanation of stochastic dominance, a technique that allows for the ranking of different alternatives (here, presence of different GM traits and their combinations) based on their overall effect on their returns (here, the yield distribution). We emphasize how it relates with decision-makers preferences in a way that is flexible enough to allow for the ranking of distributions even in the absence of detailed assumptions about utility functions. More formal treatments of stochastic dominance can be found in [18–20].

Consider, as an example, three (artificial) alternatives—*A*, *B* and *C*—with returns characterized by a stochastic variable *x* (eg, three different corn technologies, as characterized by their yield). The examples used here correspond to three probability density functions that follow a Gamma distribution (generated in Stata 12 using the -gammaden- function, with the following shape, scale and location parameters: A = (9, 0.5, 0), B = (7.5, 1, 0) and C = (2, 2, 0)).

**Fig 1:**
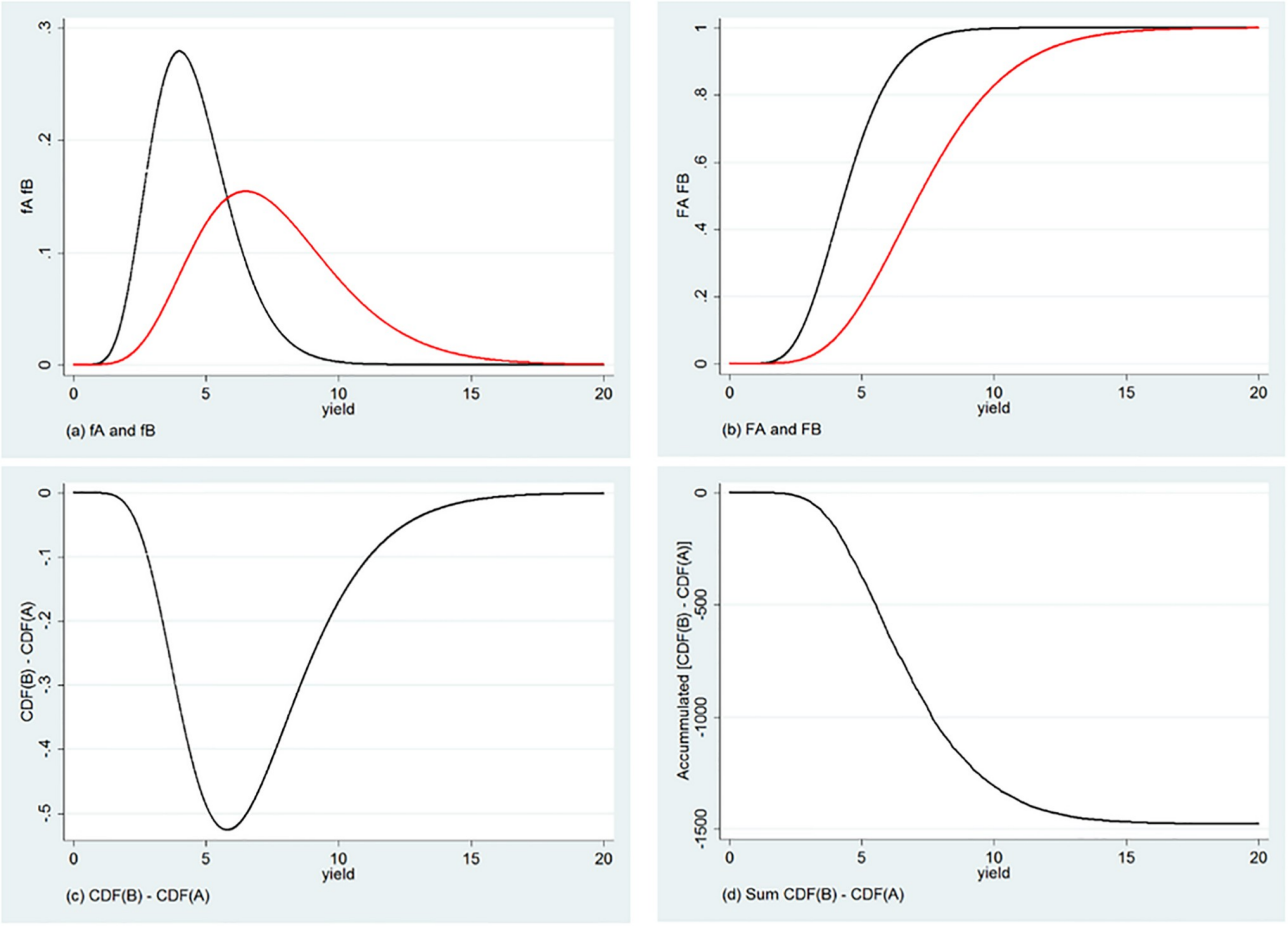
Comparing two distributions: *B* first order stochastically Dominates *A*.

As it is clear from Fig 1a, which presents the probability density functions of these two technologies (*f*_*A*_ and *f*_*B*_), a mean-variance comparison of these alternatives does not allow us to decide which one is preferable: although *B* has a higher mean, this change comes accompanied by a higher variance.

As an alternative, we can compare, for each value of *x* = *x*^⋆^, the probability of getting at most such outcome—that is, *Prob*(*x*) ≤ *x*^⋆^—which is equivalent to describing these technologies through their respective empirical Cumulative Distribution Functions (*F*_*A*_ and *F*_*B*_), presented in Fig 1b. Because *F*_*B*_ is always to the right of *F*_*A*_, then *B* is always preferable to *A* by a decision-maker who prefers more to less. It is in that sense that we say that *F*_*A*_ First Order stochastically Dominates (FOD) *F*_*B*_. This ranking is also reflected in the sign of the difference between the two CDFs, which is the same for all values of *x*, as shown in Fig 1c.

**Fig 2:**
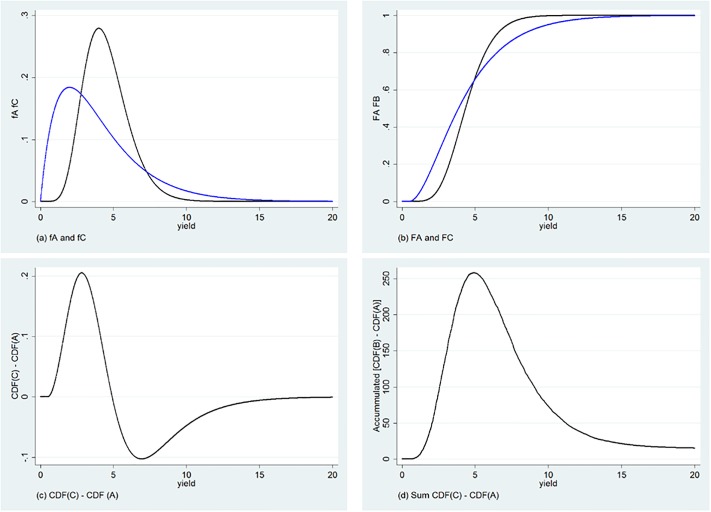
Comparing two distributions: *A* second order stochastically Dominates *C*.

It is not possible to use these criteria to rank the two distributions *A* and *C*: as it is clear from Fig 2b, there is a point where the two empirical CDFs cross (and at which the difference in CDFs changes sign—see Fig 2c). However, as long as the *accumulated* differences between CDFs does not change sign—as in Fig 2d—it is still possible to rank the two distributions under the additional assumption that the decision maker is risk averse, and willing to trade-off the additional variability (that generates the crossing between CDFs) with the overall movement to the right of the preferred CDF. It is in this sense that we say that *F*_*C*_ Second Order stochastic Dominates (SOD) *F*_*A*_.

Although the graphical representation of Third Order stochastic Dominance (TOD) becomes cumbersome, it follows a similar intuition. When ranking based on a SOD criteria (and the assumption of risk aversion) is not possible, it may still be the case that shifts in the probability distribution function that place higher probability into higher values of *x* allow us to rank the different alternatives if they lead to reductions in the probability of very low values of *x* (ie, higher skewness) and decision-makers are averse to downside risk. Finally, one must note that FOD implies SOD, and SOD implies higher orders of stochastic dominance [19].

## GM corn and yield risk: Data and empirical results

We use data from the results of experimental field trials of corn hybrids, independently run by the State Agricultural Extension Services of universities in the ten most important corn-producing states in the United States, in the first 13 years since the commercial introduction of GM corn in 1997. The data report yield for 163,941 observations of 14,614 hybrids, at 339 locations, with information about the genetic make-up of each hybrid (its traits and the degree of stacking of traits), rich detail on agronomic practices (yield, seeding rate, nitrogen application, cultivation type, previous crop, whether the trial is early or late, and whether or not irrigation was applied) and environmental conditions (soil type, rainfall and average minimum and maximum temperatures for each of the months April to September), all of which may potentially influence yield and its variability. Some summary statistics are presented in Table 1. A more complete description of the data and their sources can be found in the S1 Appendix.

**Table 1:** Summary statistics.

Variable	Definition	Mean	Std. Dev.	Min	Max
Yield	Bushels per acre of shelled grain (56lb/bu)adjusted to a moisture content of 15.5%	181.33	39.96	1	317
Conventional	= 1 if conventional hybrids (base case)	0.43	0.49	0	1
CB	= 1 if hybrid has corn borer resistant trait only	0.21	0.41	0	1
RW	= 1 if hybrid has corn rootworm resistant trait only	0.00	0.05	0	1
HT	= 1 if hybrid has herbicide tolerant trait only	0.04	0.20	0	1
CBHT	= 1 if hybrid has both corn borer resistant and herbicide tolerant traits	0.11	0.32	0	1
RWHT	= 1 if hybrid has both corn rootworm resistant and herbicide tolerant traits	0.01	0.10	0	1
CBRW	= 1 if hybrid has both corn borer resistant and corn rootworm resistant traits	0.01	0.09	0	1
CBRWHT	= 1 if hybrid is at least triple stacked with corn borer resistant, corn rootworm resistant and herbicide tolerant traits	0.19	0.39	0	1
Plant density	Plant density in thousands of kernels per acre	29.40	3.48	8.65	43.47
No or min till	= 1 if no or minimum till	0.08	0.28	0	1
Conventional till	= 1 if conventional soil preparation	0.92	0.28	0	1
Irrigated	= 1 if crop grown with irrigation	0.13	0.34	0	1
Dryland	= 1 if crop grown without irrigation	0.87	0.34	0	1
Early	= 1 if an early trial	0.23	0.42	0	1
Late	= 1 if a late trial (base case)	0.77	0.42	0	1
Soybean	= 1 if soybean was the previous crop in the rotation (base case)	0.83	0.38	0	1
Corn	= 1 if corn was the previous crop in the rotation	0.08	0.27	0	1
Wheat	= 1 if wheat was the previous crop in the rotation	0.05	0.22	0	1
Alfalfa	= 1 if alfalfa was the previous crop in the rotation	0.01	0.12	0	1
Other	= 1 if other crop was the previous crop in the rotation	0.03	0.16	0	1
Silt loam	= 1 if silt loam soil (base case)	0.56	0.50	0	1
Clay	= 1 if clay soil	0.02	0.15	0	1
Silty clay loam	= 1 if if silty clay loam soil	0.18	0.39	0	1
Clay loam	= 1 if clay loam soil	0.10	0.30	0	1
Loam	= 1 if loam soil	0.08	0.27	0	1
Sandy loam	= 1 if sandy loam soil	0.05	0.23	0	1
Sand	= 1 if sand	0.00	0.06	0	1
Nitrogen (lbs /ac)	Nitrogen application in lbs per acre	141.54	76.92	0	380
Nitrogen not reported	= 1 if nitrogen use was not reported	0.15	0.36	0	1

These data have several characteristics that make their use especially appropriate for our objective of analyzing the effect of GM traits on yield variability. The first is that we are able to include in our analysis data for corn producing areas that are outside the traditional Corn Belt (namely South Dakota, Kansas, Nebraska, Minnesota, northern Wisconsin, Missouri and parts of Ohio). We can, therefore, analyze the relative performance of a large number of GM and conventional hybrids under a wider range of production conditions than in previous analyses. The broad geographical coverage reflected in our data is an advantage, since, as noted by [16], the Corn Belt is largely defined by relatively low level of yield risk for corn. As a result, we are able to measure the effect of GM traits on the entire yield distribution where variability may matter most. A similar reasoning is presented in [15], where different regions of Wisconsin are compared.

An additional advantage is that, similarly to some previous work [14, 15] we have trial level data. This allows us to avoid the problems of using data at county or state level, which are likely to underestimate farm level risk (for example, [16] find that average yield coefficients of variation measured at the farm level are more than double those measured at state level and more than three times those measured at national level) while its experimental nature makes it particularly suited to the use of the approaches identified in the previous section, as it allows us to address the well-recognized identification problems associated with the estimation of production functions [21–23] and to identify the inclusion of GM traits as the source of difference in yield distributions. Finally, the availability of a large dataset allows us to use the stochastic dominance approach, something that is not always possible since estimation of stochastic relationships between crop yield and input use often requires large amounts of data [4]. However, one important shortcoming of this data (that we share with the *ex ante* analyses) is that we do not have data on production costs, and as such as we are not able to estimate the effect of GM traits on input costs and profits. Similarly, we are not able to evaluate the impact of GM technologies on other aspects such as health or the environment. These are likely important (see the discussion in [24]) but likewise require data at the producer level.

We take advantage of the richness of the data to control for a wide variety of agronomic practices, location characteristics and weather conditions. Because we have multiple observations for the same hybrids and we are mostly interested in obtaining consistent estimates of conditional yield, we estimate a (linear) production function with hybrid fixed effects, which can be written as

$$\begin{array}{c} y_{it}=\alpha_{i}+X_{it}^{\prime }\beta +u_{it} \end{array}$$ (1)

where *y*_*it*_ is the yield of hybrid *i* in year *t*, *X*_*it*_ is the set of covariates presented in Table 1, together with a set of interactions between location and time fixed effects that account, respectively, for any location and year specific occurrences that were not accounted for elsewhere in the data (including pest pressure and associated pesticide use), *α*_*i*_ is the unobserved (fixed) effect of the underlying germplasm and *u*_*it*_ is the idiosyncratic error term. We also restrict the sample to hybrids for which we have at least five trials (147,790 observations from 8,423 hybrids). We can then use these estimates to identify the effect of GM traits on the entire distribution of conditional yield.

**Fig 3:**
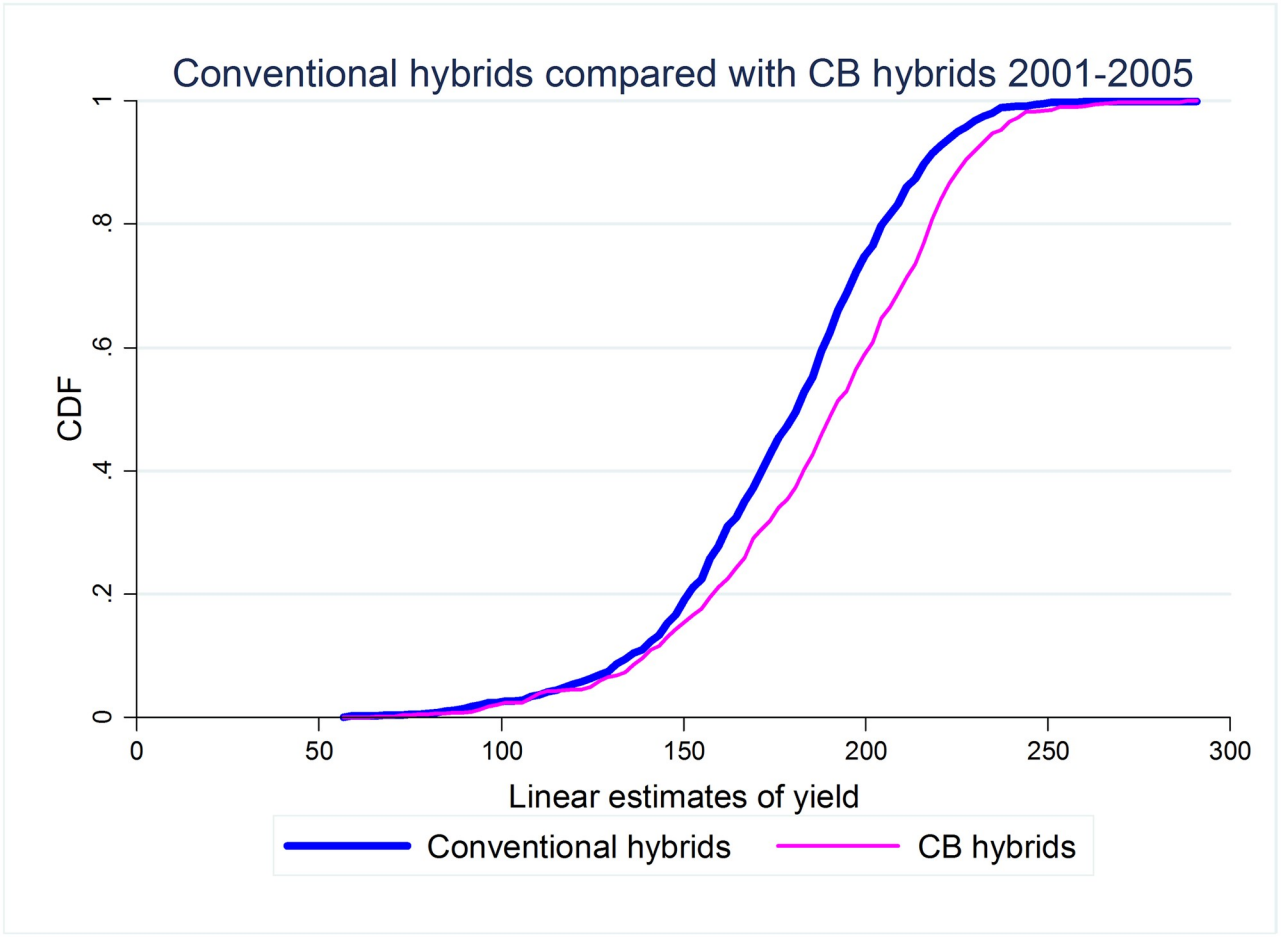
Stochastic dominance: CB vs. conventional hybrids, 2001-2005.

**Fig 4:**
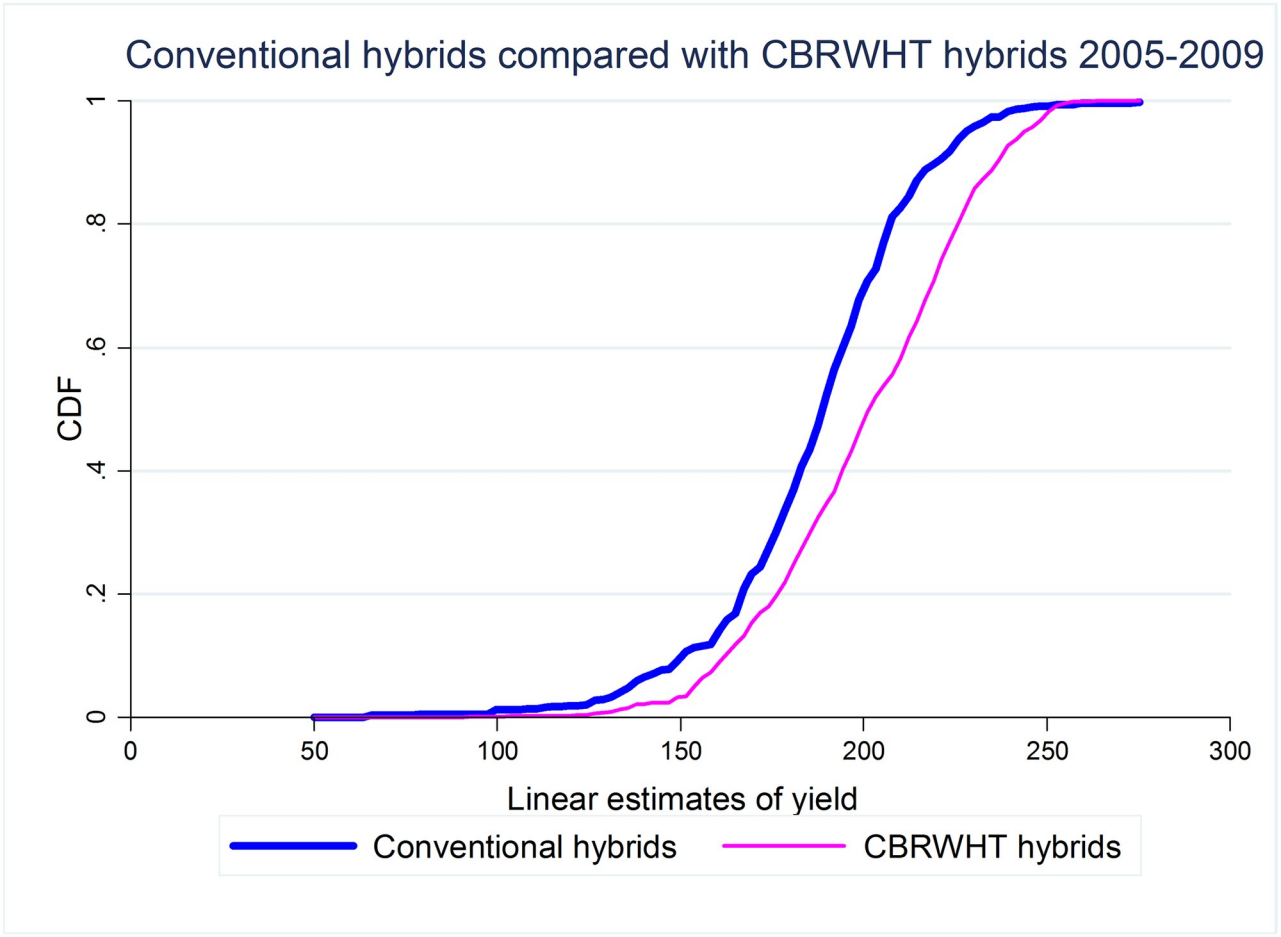
Stochastic dominance: Triple stacked vs. conventional hybrids, 2005-2009.

The CDFs of the conditional yield for both conventional hybrids and the two most frequently trialed GM hybrids are shown in Figs 3 and 4, while the CDFs of the other hybrids for which we have information are presented as Figs B to E in S1 Appendix. There are too few trials of RW hybrids for the graph of their distribution to be meaningful, hence we do not present the impact of this trait in isolation. In comparing these distributions, we restrict the analysis for each trait to the sub-periods that maximize the overlap between the number of hybrids with that specific trait and conventional hybrids, and hence also account for the timing of the introduction of the various GM traits (and associated technological change). Additional information on the distribution of trials during this period can be found in the S1 Appendix.

As explained in the previous section, if the CDF of GM hybrids in the sample lies everywhere to the right of the CDF of conventional hybrids in the sample, then GM hybrids first order dominate (FOD) conventional hybrids and, under the assumption that decision makers prefer higher yields to lower yields, we can say that GM hybrids are welfare improving. As it is obvious from these figures, GM hybrids do not first order dominate conventional hybrids. This is also true for the other hybrids, as can be inferred from Figs B to E in S1 Appendix.

We base our judgments about higher orders of stochastic dominance on the empirical likelihood ratio tests presented in [25]. Their approach involves comparing stochastic dominance curves at different order (in our case, first, second and third) and estimating the crossing points (or critical values) of the dominance curves at which there is a reversal of the ranking of the curves (if any). In addition, we test whether any difference between the corresponding distributions is statistically significant. We implement these tests using the Distributive Analysis Stata Package (DASP): Version 2.3 [26]. The results are summarized in Table 2.

**Table 2:** Stochastic dominance results.

Trait	Period	# trials GM hybrids	# trials conventional hybrids	FOD	Range over which GM hybrids dominate	% GM trials below lower crossing	% GM trials above upper crossing	SOD	TOD
CB	2001-2005	18706	22997	No	113.84–286.44	7.25	0.14	Yes	–
RW	2004-2006	349	7457	No	multiple cross	–	–	No	No
HT	2002-2007	3667	16112	No	multiple cross	–	–	No	No
CBHT	1999-2009	16622	42799	No	70.5-262.76	1.23	0.6	No	Yes
CBRW	2005-2008	1127	5501	No	0-236.61	0	5.18	Yes	–
RWHT	2005-2008	1336	5501	No	0-253.74	0	1.48	Yes	–
CBRWHT	2005-2009	27309	5831	No	0-254.14	0	4.51	Yes	–

As it can be seen from the results, no GM hybrid first order dominates conventional hybrids, confirming our earlier graphical analysis. We also present the range of yields over which the GM hybrids dominate conventional hybrids. In general, crossing points correspond to very low or very high yields, suggesting that some conventional hybrids may perform better than GM hybrids under extreme (negative or positive) conditions. However, this statement needs to be weighted by two considerations. The first is that, in most cases, GM hybrids dominate conventional hybrids for very low yields, suggesting that GM hybrids reduce downside risk (for example, CBRWHT hybrids dominate conventional hybrids for all values of yield up to 254.14 bu/acre). The second consideration concerns the very low frequency of observations for which conventional hybrids dominate GM hybrids which ranges from a minimum of 1.29% (for CBHT hybrids) to a maximum of 7.39% (for CB hybrids), in the latter mostly limited to hybrids trialed in the earlier part of the period under study.

There are two potential explanations for this last result. The first is yield drag during earlier stages of the development of this technology. The second is that GM traits were introduced into worse varieties during the same period. Disentangling these two competing explanations is impossible in our data, but the results in [27], for Wisconsin, suggest that selectivity may not play a major role in these differences.

The analysis of higher orders of stochastic dominance shows that hybrids with trait combinations found in the large majority of the varieties being trialed second order stochastically dominate conventional hybrids. This is true for those hybrids that include only corn borer (the first to be commercially released), corresponding to over one third of all GM hybrids trialed, and for triple stacked hybrids which correspond to another third of the GM hybrids trialed and that largely dominate the most recent trials. Finally, our results show that double stacked hybrids containing the corn borer and herbicide tolerance traits (CBHT), which represent approximately 20% of the GM hybrids under trial, third order dominate conventional hybrids. Hence, it seems that under relatively weak assumptions regarding producers’ preferences (namely, that producers are risk averse or that they are averse to downside risk), the most commonly trialed GM hybrids would be preferred to conventional hybrids.

## Conclusion

Although production risk is a fundamental aspect of agriculture, there has been so far very limited evidence of the yield risk effects associated with the introduction of GM traits in corn. In this article we use a large, rich dataset collated from the results of experimental hybrid corn trials independently conducted by ten university extension services in the most important corn-producing states in the USA over 13 years to investigate the effects on the entire distribution conditional yield.

The use of stochastic dominance allows us to rank the changes in the yield distribution under very weak assumptions regarding producers’ preferences. We find that GM hybrids do not first order stochastically dominate conventional hybrids which, given earlier results regarding the effect of GM traits on mean yield, would suggest that the presence of GM traits increases yield variance. However, in almost 95% of the trials over this period, GM hybrids either second-order or third-order dominate conventional hybrids. As a result, we can conclude that the effect of increased mean yield more than compensates for any increase in variability in terms of producers’ welfare under relatively weak assumptions about their preferences. These results contribute to explain the rapid adoption of this technology in the USA.

## Supporting information

S1 Appendix Data description & sources. Additional Figures.
(PDF)
