# Supplementary material for: Genetic modification and yield risk: A stochastic dominance analysis of corn in the USA
Source: PLoS One. 2019 Oct 10;14(10):e0222156. doi: 10.1371/journal.pone.0222156 (PMC6786559; doi:10.1371/journal.pone.0222156)
Supplement: S1 Appendix — Additional Figures. (PDF) [file pone.0222156.s001.pdf]

Genetic Modification and yield risk: a stochastic dominance analysis of corn in the USA – Supplementary Information

Elizabeth Nolan[1], Paulo Santos[2*]

1 Affiliation School of Economics, The University of Sydney, Sydney, NSW, Australia

2 Affiliation Dept Economics, Monash University, Caulfield, Vic, Australia

* paulo.santos@monash.edu

Data

Summary statistics for the variables included in the analysis are provided in Table **??**, in the text.

Trials by year and state: The number of observations by year and state is shown in Table A1. Number of trials by GM category is shown in Table A2.

Table A1. Number of Trials of Hybrids by Year and State

Year	Illinois	Indiana	Iowa	Kansas	Minnesota	Missouri	Nebraska	Ohio	S Dakota	Wisconsin	Total
1997	1189	981	3693	642	823	1190	1139	1004	535	2146	13342
1998	1069		3245	668	789	308	1169	955	590	2063	10856
1999	2095		3409	621	993	1223	1149	967	634	2159	13250
2000	1810	1626	3575	555	985	334	1332	853	556	1997	13623
2001	1739	1710	3321	671	859	1168	1087	844	593	1767	13759
2002	1302	1629		505	697	1201	1010	844	481	1765	9434
2003	1630	1155		466	735	1389	996	888	522	1797	9578
2004	2005	1341		672	931	1468	1149	1010	731	1818	11125
2005	1925	1471	2214	679	836	1479	1043	941	494	1803	12885
2006	1816	1196	2607	702	1190	1825	1023	838	640	1682	13519
2007	1778	1160	2810	932	1296	1529	1352	1215	588	2205	14865
2008	2020	1470	2587	1029	1039	1585	1201	1053	472	1779	14235
2009	1565	1241	2397	1028	940	1589	1185	1435	420	1669	13469
Total	21943	14980	29858	9170	12113	16288	14835	12847	7256	24650	163940

Dependent variable: Grain yields are reported as bushels per acre of shelled grain (56 lb/bu) adjusted to a moisture content of 15.5%.

Agronomic variables:

Table A2. Number of Trials by Year and GM category

year	Conventional	CB	RW	HT	CBHT	CBRW	RWHT	CBRWHT	Total GM	Total
1997	12906	408		20	8				436	13342
1998	9683	1048		78	53				1179	10862
1999	8694	3589		705	269				4563	13257
2000	9730	3289		445	151				3885	13615
2001	9880	2910		671	301				3882	13762
2002	5579	2755		533	567				3855	9434
2003	3653	4319	47	497	1047		8	7	5925	9578
2004	3133	5242	219	672	1713	25	77	44	7992	11125
2005	3633	4979	122	925	2678	194	107	247	9252	12885
2006	1955	3031	149	1123	4466	462	412	1912	11564	13519
2007	589	1517	24	916	4387	433	501	6498	14276	14865
2008	446	666	9	608	1881	200	425	9999	13788	14234
2009	476	246	2	378	1544	58	114	10645	12987	13463
Total	70357	33999	572	7571	19065	1372	1653	29352	93584	163941

Early or late: Most states conduct early and late maturity trials, but in some cases the distinction was not made until the late 1990s or early 2000s. Some states still do not make a distinction. If there is not a specific statement that the trial is early season we have assumed that it is late. Nebraska reports on mid trials in some years but we classified these as late. A dummy variable is used to indicate an early trial.

Irrigated or dryland: Missouri, Nebraska, Kansas, and Wisconsin conduct irrigated trials, and a dummy variable is included to indicate whether a trial is irrigated.

Minimum or no tillage compared with conventional tillage: Type of cultivation is reported in some detail and it was not possible to account for all of the variations. A dummy variable has been used to indicate minimum or no till preparation, but only where this is explicitly stated. The default variable is conventional and every other type of cultivation is included in this category.

Soil type: Seven soil types are identified by dummy variables, with silt loam as the default soil. The only state that does not report soil type is Minnesota and we used the coordinates for each trial location and the Soil Web Survey of the USDA Natural Resources Conservation Service (USDA Natural Resources Conservation Service 2010) to identify the dominant soil type in that location.

Rotation: Previous crop is reported for most locations. However, Illinois does not report on rotation, and, in a small number of other locations in other states, the rotation is omitted. As soybean is the usual rotation crop, we assumed that this is the previous crop where it was missing. Dummy variables have been included for corn,

wheat, alfalfa, and other, with soybean as the base case.

Plant density: We used final plant population (in thousands) where this is reported. Where final plant population was not reported we have substituted seeding rate. Although the two variables are not identical, the order of magnitude is in general similar.

Fertilizer: We have data on nitrogen fertilizer application in lbs/acre for most states. However, Illinois started to report fertilizer application rates only in 2000. Iowa does not report fertilizer rates. We included a zero value for the missing observations. To differentiate between cases where nitrogen use was reported as zero, and the missing observations we created a dummy variable with a value of 1 indicating "Nitrogen not reported". Although some states do report phosphorus and potassium application, others do not, and we did not include these fertilizers in our analysis.

Pesticides and herbicides: It would have been useful to include pesticide and herbicide application rates. However the variety of different combinations that are possible and that have been used over the past 13 years is too large. We have assumed that the trials are conducted so as to eliminate pest and weed infestations.

Climatic variables - rainfall and average maximum and minimum temperatures: In most cases the trial reports include rainfall for the growing months. If not (for example, for Ohio and Iowa), there is generally a very good network of weather stations and it was possible to extract monthly rainfall from their databases (Iowa Environmental Mesonet 2009; OARDC 2009). For those states which do not report specific rainfall figures (Nebraska includes column charts, and Minnesota does not report rainfall) we used the data provided by the PRISM Climate Group at the University of Oregon (PRISM Climate Group Oregon State University 2009), which allows for monthly rainfall, minimum and maximum temperatures to be extracted based on latitude and longitude coordinates. Some universities have reported rainfall May-September, others April-August and others April-September. We filled the gaps for the months April-September from the PRISM database. As temperature is likely to be less local than rainfall, we extracted minimum and maximum monthly temperatures during the period April- September from the PRISM database.

Other variables

Location where trial conducted: We have details of the location where the trial was conducted. The locations are not necessarily exactly correlated with the included site

characteristics. The trials may not be at exactly the same site each year, or may be at different farms or sites in the immediate area. At some locations trials are held on more than one soil type.

Interaction term for Crop Reporting District (CRD) by year: It is likely that there are some factors that are variable by year and by location. In particular, we do not include variables related to pest pressure or chemical use, because it was too difficult to obtain consistent information across states. The CRD by year interaction terms are therefore included to account for different chemical usage practices and different degrees of pest infestation. The weather data is by location, not by CRD. Therefore weather is not exactly correlated with the CRD × year interaction term included in the estimation.

GM traits and stacking of traits: We have details of the GM traits associated with each hybrid. We identified the presence of these traits using dummy variables, and created dummy variables to indicate the combinations of traits where traits are stacked. The base case is no GM traits (conventional hybrids). The number of trials by year and by category of GM traits for the whole dataset can be found in Table A1 and Fig A.

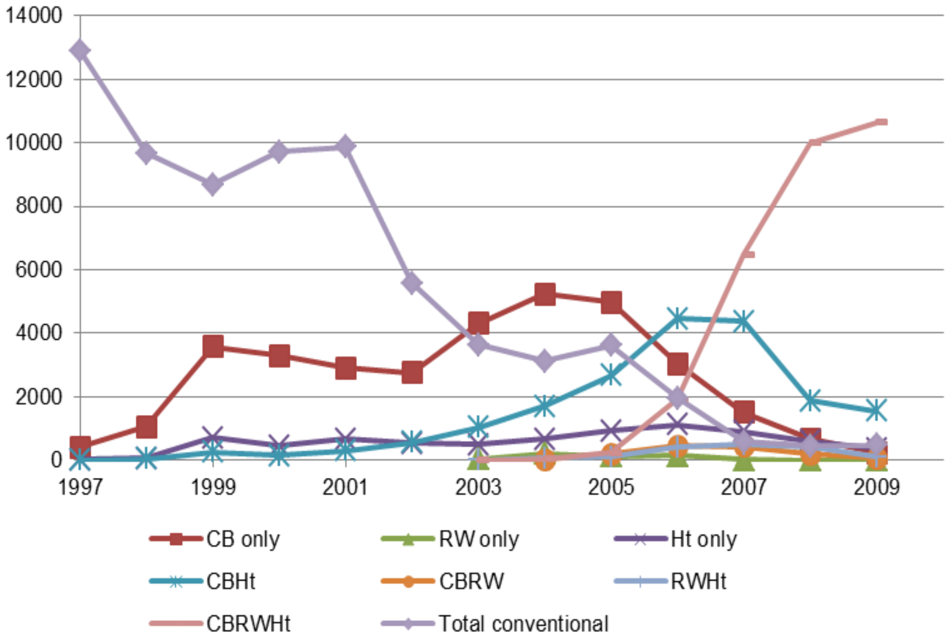

Fig A. Trials by GM category

Hybrid identifiers: The trial reports provide the name of the company submitting the hybrid for trial, the name of the hybrid, and, since the introduction of genetically

modified hybrids, the GM traits associated with each hybrid. Since some quite different hybrids have the same number, we have identified each separate hybrid by combining the name of the submitting company and the name of the hybrid. It is this variable that we have used to create hybrid identifiers.

Where the hybrid number is the same, and the submitting company has changed, but is known to be affiliated with the previous submitting company, we have considered the hybrids to be identical. In some cases a hybrid will have the same name, but a different submitting company in consecutive years. For example, Keltgen, Lynks and Mycogen all submitted a hybrid with the same name in different years in the mid-1990s. Mycogen acquired Keltgen and Lynks in the early to mid-1990s, so we have assumed that these hybrids are in fact the same, and have renamed the hybrid identifier accordingly. Kruger Seed Company has at times submitted seed under the company names Kruger, KSC/Challenger, Circle and Desoy.

Missing data

We relied on the cooperation of the various extension services to obtain copies of those reports which are not available online, and some records are not complete.

Iowa has the longest history of testing but records are incomplete. Records are complete from 2005. Professor Joe Lauer (UW-Madison) kindly provided us with data for individual locations for 1997-2001. The years 2002-2004 are lost. Even though we only have ten years of data for Iowa, the number of trials is substantial.

Cultivation type and rotation were not reported by Ohio for 1998-2002 but the locations and agronomic practices for other years are consistent, hence we assumed that the same cultivation methods and rotation decisions were made.

In some years Indiana reports only regional average yields, so we omitted those years and those locations where individual site results are not reported. This means that we have no entries for 1998-1999, and limited entries for 1997.

The University of Missouri is missing reports for 1998 and 2000, but some of the 1998 and 2000 results are reported in the following years' reports and we included those results.

References

Iowa Environmental Mesonet. 2009. IEM "Climodat" Reports. Available at `http://mesonet.agron.iastate.edu/climodat/index.phtml`. Accessed on various

dates in 2009.

OARDC. 2009. OARDC Weather System. Available at http://www.oardc.ohio-state.edu/newweather/. Accessed on various dates in 2009.

PRISM Climate Group Oregon State University. 2009. PRISM Data Explorer. Available at http://gisdev.nacse.org/prism/nn/index.phtml. Accessed on various dates in 2009 and 2010.

USDA Natural Resources Conservation Service. 2010. Web Soil Survey. Available at http://websoilsurvey.nrcs.usda.gov/app/. Accessed on 22 January, 2010.

Data Sources, by State and by Year

Illinois

Department of Crop Sciences University of Illinois at Urbana-Champaign 1997. Corn Hybrid Variety Trials in Illinois - 1997 Results. Department of Crop Sciences, University of Illinois at Urbana-Champaign.

Department of Crop Sciences University of Illinois at Urbana-Champaign 1998. Corn Hybrid Variety Trials in Illinois - 1998 Results. Department of Crop Sciences, University of Illinois at Urbana-Champaign.

Department of Crop Sciences University of Illinois at Urbana-Champaign 1999. Corn Hybrid Variety Trials in Illinois - 1999 Results. Department of Crop Sciences, University of Illinois at Urbana-Champaign.

Department of Crop Sciences University of Illinois at Urbana-Champaign 2000. Corn Hybrid Variety Trials in Illinois - 2000 Results. Department of Crop Sciences, University of Illinois at Urbana-Champaign.

Department of Crop Sciences University of Illinois at Urbana-Champaign 2001. Corn Hybrid Variety Trials in Illinois - 2001 Results. Department of Crop Sciences, University of Illinois at Urbana-Champaign.

Department of Crop Sciences University of Illinois at Urbana-Champaign 2002. Corn Hybrid Variety Trials in Illinois - 2002 Results. Department of Crop Sciences, University of Illinois at Urbana-Champaign.

Department of Crop Sciences University of Illinois at Urbana-Champaign 2003. Corn Hybrid Variety Trials in Illinois - 2003 Results. Department of Crop Sciences, University of Illinois at Urbana-Champaign.

Department of Crop Sciences University of Illinois at Urbana-Champaign 2004. Corn Hybrid Variety Trials in Illinois - 2004 Results. Department of Crop Sciences, University of Illinois at Urbana-Champaign.

Department of Crop Sciences University of Illinois at Urbana-Champaign 2005. Corn Hybrid Test Results in Illinois – 2005. Department of Crop Sciences, University of Illinois at Urbana-Champaign.

Joos, D., R. Esgar, B. Henry, E. Nafziger, and C. Smyth. 2006. Corn Hybrid Test Results in Illinois - 2006 Crop Sciences Special Report 2006-03. Department of Crop Sciences, University of Illinois at Urbana-Champaign.

Joos, D., R. Esgar, B. Henry, E. Nafziger, and C. Smyth. 2007. Corn Hybrid Test Results in Illinois - 2007 Crop Sciences Special Report 2007-03. Department of Crop Sciences, University of Illinois at Urbana-Champaign.

Joos, D., R. Esgar, B. Henry, E. Nafziger, and C. Smyth. 2008. Corn Hybrid Test Results in Illinois - 2008 Crop Sciences Special Report 2008-03. Department of Crop Sciences, University of Illinois at Urbana-Champaign.

Joos, D., R. Esgar, B. Henry, E. Nafziger, and C. Smyth. 2009. Corn Hybrid Test Results in Illinois - 2009. Crop Sciences Special Report. 2009-03. Department of Crop Sciences, University of Illinois at

Urbana-Champaign.

Indiana

DeVillez, P. and W. Foster. 1997. Performance of Commercial Dent Corn Hybrids in Indiana, 1994-1997. Bulletin No. B-757. Department of Agronomy, Purdue University.

DeVillez, P. and W. Foster. 1998. Performance of Commercial Dent Corn Hybrids in Indiana, 1995-1998 Bulletin No. B-776. Department of Agronomy, Purdue University.

DeVillez, P. and W. Foster. 1999. Performance of Commercial Dent Corn Hybrids in Indiana, 1996-1999. Bulletin No. B-785. Department of Agronomy, Purdue University.

DeVillez, P. and W. Foster. 2000. Performance of Commercial Dent Corn Hybrids in Indiana, 1998-2000. Bulletin No. B-797. Department of Agronomy, Purdue University.

DeVillez, P. and W. Foster. 2001. Performance of Commercial Dent Corn Hybrids in Indiana, 2001 Bulletin No. B-806. Department of Agronomy, Purdue University.

DeVillez, P. and W. Foster. 2002. Performance of Commercial Dent Corn Hybrids in Indiana, 2001-2002 Bulletin No. B-816. Department of Agronomy, Purdue University.

DeVillez, P. and W. Foster. 2003. Performance of Commercial Dent Corn Hybrids in Indiana, 2001-2003. Bulletin No. B-823. Department of Agronomy, Purdue University.

DeVillez, P. and W. Foster. 2004. Performance of Commercial Dent Corn Hybrids in Indiana, 2002-2004. Bulletin No. . B-17508. Department of Agronomy, Purdue University.

DeVillez, P. and W. Foster. 2005. Performance of Commercial Dent Corn Hybrids in Indiana, 2003-2005. Bulletin No. B-17818. Department of Agronomy, Purdue University.

DeVillez, P., W. Foster, and P. Lorton. 2006. 2006 Purdue Corn and Soybean Performance Trials. Department of Agronomy, Purdue University.

DeVillez, P., W. Foster, and P. Lorton. 2007. 2007 Purdue Corn and Soybean Performance Trials. Department of Agronomy, Purdue University.

DeVillez, P. and W. Foster. 2008. 2008 Purdue Corn and Soybean Performance Trials. Department of Agronomy, Purdue University.

DeVillez, P. and W. Foster. 2009. 2009 Purdue Corn and Soybean Performance Trials. Department of Agronomy, Purdue University

Iowa

University Extension Iowa State University 1999. 1999 Iowa Crop Performance Test - Corn, Iowa Farmer Today (supplement), 11 December.

University Extension Iowa State University 2000. 2000 Iowa Crop Performance Test - Corn, Iowa Farmer Today (supplement), 16 December.

Crop Testing Iowa State University. 2005. 2005 Iowa Crop Performance Test - Corn. University Extension, Iowa State University.

Crop Testing Iowa State University 2006. 2006 Iowa Crop Performance Test - Corn, University Extension, Iowa State University.

Crop Testing Iowa State University. 2007. 2007 Iowa Crop Performance Test - Corn. University Extension, Iowa State University.

Crop Testing Iowa State University. 2008. 2008 Iowa Crop Performance Test - Corn. University Extension, Iowa State University.

Crop Testing Iowa State University. 2009. 2009 Iowa Crop Performance Test - Corn. University Extension, Iowa State University.

Kansas

Roozeboom, K. 1997. 1997 Kansas Performance Tests with Corn Hybrids. Report of Progress. 796. Kansas State University Agricultural Experiment Station and Cooperative Extension Service.

Roozeboom, K. 1998. 1998 Kansas Performance Tests with Corn Hybrids. Report of Progress. 822. Kansas State University Agricultural Experiment Station and Cooperative Extension Service.

Roozeboom, K. 1999. 1999 Kansas Performance Tests with Corn Hybrids. Report of Progress. 843. Kansas State University Agricultural Experiment Station and Cooperative Extension Service.

Roozeboom, K. 2000. 2000 Kansas Performance Tests with Corn Hybrids. Report of Progress. 869. Kansas State University Agricultural Experiment Station and Cooperative Extension Service.

Roozeboom, K. 2001. 2001 Kansas Performance Tests with Corn Hybrids. Contribution No. 02-173-S. Kansas State University Agricultural Experiment Station and Cooperative Extension Service.

Roozeboom, K. 2002. 2002 Kansas Performance Tests with Corn Hybrids. Report of Progress. 899. Kansas State University Agricultural Experiment Station and Cooperative Extension Service.

Roozeboom, K. 2003. 2003 Kansas Performance Tests with Corn Hybrids. Contribution No. 04-165-S from the Kansas Agricultural Experiment Station. Kansas State University Agricultural Experiment Station and Cooperative Extension Service.

Roozeboom, K. 2004. 2004 Kansas Performance Tests with Corn Hybrids. Contribution No. 05-145-S from the Kansas Agricultural Experiment Station. Kansas State University Agricultural Experiment Station and Cooperative Extension Service.

Roozeboom, K. 2005. 2005 Kansas Performance Tests with Corn Hybrids. Report of Progress. 949. Kansas State University Agricultural Experiment Station and Cooperative Extension Service.

Lingenfelser, J. 2006. 2006 Kansas Performance Tests with Corn Hybrids. Report of Progress. 968. Kansas State University Agricultural Experiment Station and Cooperative Extension Service.

Lingenfelser, J. 2007. 2007 Kansas Performance Tests with Corn Hybrids. Report of Progress. 983. Kansas State University Agricultural Experiment Station and Cooperative Extension Service.

Lingenfelser, J. 2008. 2008 Kansas Performance Tests with Corn Hybrids. Report of Progress. 1000. Kansas State University Agricultural Experiment Station and Cooperative Extension Service.

Lingenfelser, J. 2009. 2009 Kansas Performance Trials with Corn Hybrids. Report of Progress. 1019. Kansas State University Agricultural Experiment Station and Cooperative Extension Service.

Minnesota

Hicks, D., T. Hoverstad, A. Kanmanzi, G. Nelson, and P. Porter. 1997. Grain Corn Variety Trials: For Crop production 1998. MR. 7126. Minnesota Agricultural Experiment Station, University of Minnesota.

Sheaffer, C., D. Hicks, T. Hoverstad, D. Swanson, and J. Halgerson. 1998. Grain Corn Variety Trials: For Crop production 1999. Report No. ES-WW-7343. Minnesota Agricultural Experiment Station, University of Minnesota.

Minnesota Agricultural Experiment Station University of Minnesota 1999. 1999 Varietal Trials Results: Corn Grain, Minnesota Agricultural Experiment Station, University of Minnesota.

Minnesota Agricultural Experiment Station University of Minnesota 2000. 2000 Varietal Trials Results: Corn Grain, Minnesota Agricultural Experiment Station, University of Minnesota.

Minnesota Agricultural Experiment Station University of Minnesota 2001. 2001 Varietal Trials Results: Corn Grain, Minnesota Agricultural Experiment Station, University of Minnesota.

Minnesota Agricultural Experiment Station University of Minnesota 2002. 2002 Varietal Trials Results: Corn Grain, Minnesota Agricultural Experiment Station, University of Minnesota.

Minnesota Agricultural Experiment Station University of Minnesota 2003. 2003 Varietal Trials Results: Corn Grain, Minnesota Agricultural Experiment Station, University of Minnesota.

Minnesota Agricultural Experiment Station University of Minnesota 2005. 2004 Varietal Trials Results: Corn Grain, Minnesota Agricultural Experiment Station, University of Minnesota.

Hoverstad, T., D. Hicks, G. Nelson, and S. Quiring. 2006. 2005 Varietal Trials Results: Corn Grain. Minnesota Agricultural Experiment Station, University of Minnesota.

Hoverstad, T., D. Hicks, G. Nelson, and S. Quiring. 2007. 2006 Varietal Trials Results: Corn Grain. Minnesota Agricultural Experiment Station, University of Minnesota.

Hoverstad, T., D. Hicks, G. Nelson, S. Quiring, and M. Hanson. 2008. 2007 Varietal Trials Results: Corn Grain. Minnesota Agricultural Experiment Station, University of Minnesota.

Hoverstad, T., J. Coulter, G. Nelson, S. Quiring, and M. Hanson. 2009. 2008 Varietal Trials Results: Corn Grain. Minnesota Agricultural Experiment Station, University of Minnesota.

Hoverstad, T., J. Coulter, G. Nelson, S. Quiring, and M. Hanson. 2010. 2009 Varietal Trials Results: Corn Grain Minnesota Agricultural Experiment Station, University of Minnesota.

Missouri

Minor, H., C. Morris, H. Mason, D. Knerr, R. Hasty, and G. Stafford. 1997. 1997 Missouri Crop Performance: Corn. Special Report. 484. Agricultural Experiment Station, College of Agriculture, Food and Natural Resources, University of Missouri- Columbia.

Minor, H., C. Morris, H. Mason, D. Knerr, R. Hasty, G. Stafford, and T. Fritts. 1999. 1999 Missouri Crop Performance: Corn. Special Report No. 521. Agricultural Experiment Station, College of Agriculture, Food and Natural Resources, University of Missouri- Columbia.

Wiebold, W., H. Mason, D. Knerr, R. Hasty, T. Fritts, and E. Adams. 2001. 2001 Missouri Crop Performance: Corn. Special Report No. 537. Agricultural Experiment Station, College of Agriculture, Food and Natural Resources, University of Missouri- Columbia.

Wiebold, W., H. Mason, D. Knerr, R. Hasty, T. Fritts, and E. Adams. 2002. 2002 Missouri Crop Performance: Corn. Special Report.No. 543. Agricultural Experiment Station, College of Agriculture, Food and Natural Resources, University of Missouri- Columbia.

Wiebold, W., H. Mason, D. Knerr, R. Hasty, E. Adams, D. Schwab, and S. Smothers. 2003. 2003 Missouri Crop Performance: Corn. Agricultural Experiment Station, College of Agriculture, Food and Natural Resources, University of Missouri-Columbia.

Wiebold, W., H. Mason, D. Knerr, R. Hasty, E. Adams, D. Schwab, and S. Smothers. 2004. 2004 Missouri Crop Performance: Corn. Agricultural Experiment Station, College of Agriculture, Food and

Natural Resources, University of Missouri-Columbia.

Wiebold, W., H. Mason, D. Knerr, R. Hasty, E. Adams, D. Schwab, T. Belt, S. Smothers, and B. Burdick. 2005. 2005 Missouri Crop Performance: Corn. Special Report No. 561. Agricultural Experiment Station, College of Agriculture, Food and Natural Resources, University of Missouri-Columbia.

Wiebold, W., H. Mason, D. Knerr, R. Hasty, E. Adams, D. Schwab, T. Belt, S. Smothers, and B. Burdick. 2006. 2006 Missouri Crop Performance: Corn. Special Report No. 566. Agricultural Experiment Station, College of Agriculture, Food and Natural Resources, University of Missouri-Columbia.

Wiebold, W., H. Mason, D. Knerr, R. Hasty, T. Belt, D. Schwab, B. Burdick, and J. Angotti. 2007. 2007 Missouri Crop Performance: Corn. Special Report No. 570. Agricultural Experiment Station, College of Agriculture, Food and Natural Resources, University of Missouri-Columbia.

Wiebold, W., H. Mason, T. Belt, D. Knerr, R. Hasty, D. Schwab, J. Angotti, and B. Burdick. 2008. 2008 Missouri Corn Performance Tests. Division of Plant Sciences, College of Agriculture, Food and Natural Resources, University of Missouri-Columbia.

Wiebold, W., H. Mason, D. Knerr, R. Hasty, D. Schwab, J. Angotti, and W. Schelp. 2009. Missouri 2009 Corn Performance Tests. Division of Plant Sciences, College of Agriculture, Food and Natural Resources, University of Missouri-Columbia.

Nebraska

Nelson, L., R. Klein, R. Elmore, D. Baltensperger, P. Nordquist, and C. Shapiro. 1997. Nebraska Corn Hybrid Tests 1997. Extension Circular No. 97-105-A. Institute of Agriculture and Natural Resources, University of Nebraska-Lincoln.

Nelson, L., R. Klein, R. Elmore, D. Baltensperger, C. Shapiro, and J. Krall. 1998. Nebraska Corn Hybrid Tests 1998. Extension Circular No. 98-105-A. Institute of Agriculture and Natural Resources, University of Nebraska-Lincoln.

Nelson, L., R. Klein, R. Elmore, D. Baltensperger, C. Shapiro, S. Knezevic, and J. Krall. 1999. Nebraska Corn Hybrid Tests 1999. Extension Circular No. 99-105-A. Institute of Agriculture and Natural Resources, University of Nebraska-Lincoln.

Nelson, L., R. Klein, R. Elmore, D. Baltensperger, C. Shapiro, S. Knezevic, and J. Krall. 2000. Nebraska Corn Hybrid Tests 2000. Extension Circular No. 00-105-A. Institute of Agriculture and Natural Resources, University of Nebraska-Lincoln.

Nelson, L., R. Klein, R. Elmore, D. Baltensperger, C. Shapiro, S. Knezevic, and J. Krall. 2001. Nebraska Corn Hybrid Tests 2001. Extension Circular No. 01-105-A. Institute of Agriculture and Natural Resources, University of Nebraska-Lincoln.

Nelson, L., R. Klein, R. Elmore, D. Baltensperger, C. Shapiro, S. Knezevic, and J. Krall. 2002. Nebraska Corn Hybrid Tests 2002. Extension Circular No. 02-105-A. Institute of Agriculture and Natural Resources, University of Nebraska-Lincoln.

Nelson, L., R. Klein, R. Elmore, D. Baltensperger, C. Shapiro, S. Knezevic, and J. Krall. 2003. Nebraska Corn Hybrid Tests 2003. Extension Circular No. 03-105-A. Institute of Agriculture and Natural Resources, University of Nebraska-Lincoln.

Institute of Agriculture and Natural Resources University of Nebraska-Lincoln. 2004. Nebraska Corn Hybrid Tests 2004. Extension Circular No. 04-101. Institute of Agriculture and Natural Resources, University of Nebraska-Lincoln.

Nelson, L., B. Anderson, R. Klein, R. Elmore, D. Baltensperger, C. Shapiro, S. Knezevic, and J. Krall. 2005. Nebraska Corn Hybrid Tests 2005. Extension Circular No. 101. UNL Extension, Institute of Agriculture and Natural Resources, University of Nebraska- Lincoln.

Nelson, L., B. Anderson, R. Klein, R. Ferguson, D. Baltensperger, C. Shapiro, S. Knezevic, and J. Krall. 2006. Seed Guide 2007. Extension Circular No. 101. UNL Extension, Institute of Agriculture and Natural Resources, University of Nebraska-Lincoln.

Nelson, L., B. Anderson, R. Klein, R. Ferguson, C. Shapiro, S. Knezevic, and J. Krall. 2007. Seed Guide 2008. Extension Circular No. 101. UNL Extesion, Institute of Agriculture and Natural Resources, University of Nebraska-Lincoln.

Institute of Agriculture and Natural Resources University of Nebraska-Lincoln. 2008. Seed Guide 2009. Extension Circular No. 101. UNL Extension, Institute of Agriculture and Natural Resources, University of Nebraska-Lincoln.

Regassa, T., R. Klein, B. Anderson, C. Shapiro, and J. Krall. 2009. Seed Guide 2010. Extension Circular No. 101. UNL Extension, Institute of Agriculture and Natural Resources, University of Nebraska-Lincoln.

Ohio

Jordan, D. 1990. Ohio Corn Performance Test 1990. Agronomy Department Series No. 215. Department of Agronomy, The Ohio State University.

Jordan, D. 1991. Ohio Corn Performance Test 1991. Agronomy Department Series No. 215. Department of Agronomy, The Ohio State University.

Jordan, D. 1992. 1992 Ohio Corn Performance Test. Agronomy Department Series No. 215. Department of Agronomy, The Ohio State University.

Jordan, D. 1993. 1993 Ohio Corn Performance Trials, Ohio's Country Journal (supplement), December, pp. 20-33.

Jordan, D. 1994. 1994 Ohio Corn Performance Trials, Ohio's Country Journal (supplement), December, pp. 19-30.

Jordan, D. and R. Minyo 1995. 1995 Ohio Corn Performance Trials, Ohio's Country Journal (supplement), December, pp. 21-32.

Jordan, D. and R. Minyo 1996. 1996 Ohio Corn Performance Trials, Ohio's Country Journal (supplement), December, pp. 22-33.

Jordan, D. and R. Minyo 1997. 1997 Ohio Corn Performance Test. Department of Agronomy, The Ohio State University.

Jordan, D. and R. Minyo 1998. 1998 Ohio Corn Performance Test. Department of Agronomy, The Ohio State University.

Jordan, D. and R. Minyo 1999. 1999 Ohio Corn Performance Test. Department of Agronomy, The Ohio State University.

Minyo, R., D. Jordan, and A. Geyer 2000. 2000 Ohio Corn Performance Test, Department of Agronomy, The Ohio State University.

Minyo, R., A. Geyer, P. Thomison, B. Bishop, and D. Lohnes 2001. 2001 Ohio Corn Performance Test. Department of Agronomy, The Ohio State University.

Minyo, R., A. Geyer, P. Thomison, B. Bishop, and D. Lohnes. 2002. 2002 Ohio Corn Performance Test. Horticulture and Crop Science Series No. 215. Ohio Agricultural Research and Development Center, The Ohio State University.

Minyo, R., A. Geyer, P. Thomison, B. Bishop, and D. Lohnes. 2003. 2003 Ohio Corn Performance Test. Horticulture and Crop Science Series No. 215. Ohio Agricultural Research and Development Center, The Ohio State University.

Minyo, R., A. Geyer, P. Thomison, B. Bishop, and D. Lohnes. 2004. 2004 Ohio Corn Performance Test. Horticulture and Crop Science Series No. 215. Ohio Agricultural Research and Development Center, The Ohio State University.

Minyo, R., A. Geyer, P. Thomison, B. Bishop, and D. Lohnes. 2005. 2005 Ohio Corn Performance Test. Ohio Agricultural Research and Development Center, The Ohio State University.

Minyo, R., A. Geyer, P. Thomison, B. Bishop, and D. Lohnes. 2006. 2006 Ohio Corn Performance Test. Department of Agronomy, The Ohio State University.

Minyo, R., A. Geyer, P. Thomison, B. Bishop, and D. Lohnes. 2007. 2007 Ohio Corn Performance Test. Ohio Agricultural Research and Development Center, The Ohio State University.

Minyo, R., A. Geyer, P. Thomison, B. Bishop, and D. Lohnes. 2008. 2008 Ohio Corn Performance Test. Ohio Agricultural Research and Development Center, The Ohio State University.

Minyo, R., A. Geyer, P. Thomison, B. Bishop, and D. Lohnes. 2009. 2009 Ohio Corn Performance Test. Ohio Agricultural Research and Development Center, The Ohio State University.

South Dakota

Hall, R. 1997. 1997 Crop Performance Trials: Corn. Report No. C253-97. Cooperative Extension Service, South Dakota State University.

Hall, R. and K. Kirby. 1998. 1998 Crop Performance Trials: Corn. Report No. C253-98. Cooperative Extension Service, South Dakota State University.

Hall, R. and K. Kirby. 1999. 1999 Crop Performance Trials: Corn. Report No. C253-99. Agricultural Experiment Station, South Dakota State University.

Hall, R. and K. Kirby. 2000. 2000 Crop Performance Trials: Corn. Report No. C253-00. Agricultural Experiment Station, South Dakota State University.

Hall, R. and K. Kirby. 2001. 2001 Crop Performance Trials: Corn. Report No. C253-01. Agricultural Experiment Station, South Dakota State University.

Hall, R. and K. Kirby. 2002. 2002 Crop Performance Trials: Corn. Report No. C253-02. Agricultural Experiment Station, South Dakota State University.

Hall, R. and K. Kirby. 2003. 2003 Precision Planted Performance Trials: Corn. Report No. C253-03. Agricultural Experiment Station, South Dakota State University.

Hall, R. and K. Kirby. 2004. 2004 Precision Planted Performance Trials: Corn. Report No. C253-04. Agricultural Experiment Station, South Dakota State University.

Hall, R., K. Kirby, and G. Piechowski. 2005. 2005 Precision Planted Performance Trials: Corn. Report No. C253-05. Cooperative Extension Service, South Dakota State University.

Hall, R. and K. Kirby. 2006. 2006 Precision Planted Performance Trials: Corn. Report No. C253-06. Agricultural Experiment Station, South Dakota State University.

Hall, R., K. Kirby, and J. Hall. 2007. 2007 Precision Planted Performance Trials: Corn. Report No. C253-07. Cooperative Extension Service, South Dakota State University.

Hall, R., K. Kirby, and J. Hall. 2008. 2008 Precision Planted Performance Trials: Corn. Report No. C253-08. Cooperative Extension Service, South Dakota State University.

Hall, R., K. Kirby, and J. Hall. 2009. 2009 Precision Planted Performance Trials: Corn. Report No. C 253-09. Cooperative Extension Service, South Dakota State University.

Wisconsin

Lauer, J., K. Kohn, P. Flannery, and K. Hudelson. 1997. 1997 Wisconsin Corn Hybrid Performance Trials Grain and Silage. Department of Agronomy, University of Wisconsin.

Lauer, J., K. Kohn, and P. Flannery. 1998. 1998 Wisconsin Corn Hybrid Performance Trials Grain and Silage. Department of Agronomy, University of Wisconsin.

Lauer, J., K. Kohn, and P. Flannery. 1999. 1999 Wisconsin Corn Hybrid Performance Trials Grain and Silage. Department of Agronomy, University of Wisconsin.

Lauer, J., K. Kohn, P. Flannery, and M. Kral. 2000. 2000 Wisconsin Corn Hybrid Performance Trial Results. Department of Agronomy, University of Wisconsin.

Lauer, J., K. Kohn, and P. Flannery. 2001 Wisconsin Corn Hybrid Performance Trial Results. Department of Agronomy, University of Wisconsin.

Lauer, J., K. Kohn, and P. Flannery. 2002. 2002 Wisconsin Corn Hybrid Performance Trial Results. Department of Agronomy, University of Wisconsin.

Lauer, J., K. Kohn, and P. Flannery. 2003. 2003 Wisconsin Corn Hybrid Performance Trial Results. Department of Agronomy, University of Wisconsin.

Lauer, J., K. Kohn, and P. Flannery. 2004. 2004 Wisconsin Corn Hybrid Performance Trials Grain and Silage. Department of Agronomy, University of Wisconsin.

Lauer, J., K. Kohn, and P. Flannery. 2005. 2005 Wisconsin Corn Hybrid Performance Trial Results. Department of Agronomy, University of Wisconsin.

Lauer, J., K. Kohn, and P. Flannery. 2006. 2006 Wisconsin Corn Hybrid Performance Trial Results. Department of Agronomy, University of Wisconsin.

Lauer, J., K. Kohn, and T. Diallo. 2007. 2007 Wisconsin Corn Hybrid Performance Trial Results. Department of Agronomy, University of Wisconsin.

Lauer, J., K. Kohn, and T. Diallo. 2008. 2008 Wisconsin Corn Hybrid Performance Trial Results. Department of Agronomy, University of Wisconsin.

Lauer, J.G., K. Kohn, and T. Diallo. 2009. 2009 Wisconsin Corn Hybrid Performance Trial Results. Department of Agronomy, University of Wisconsin.

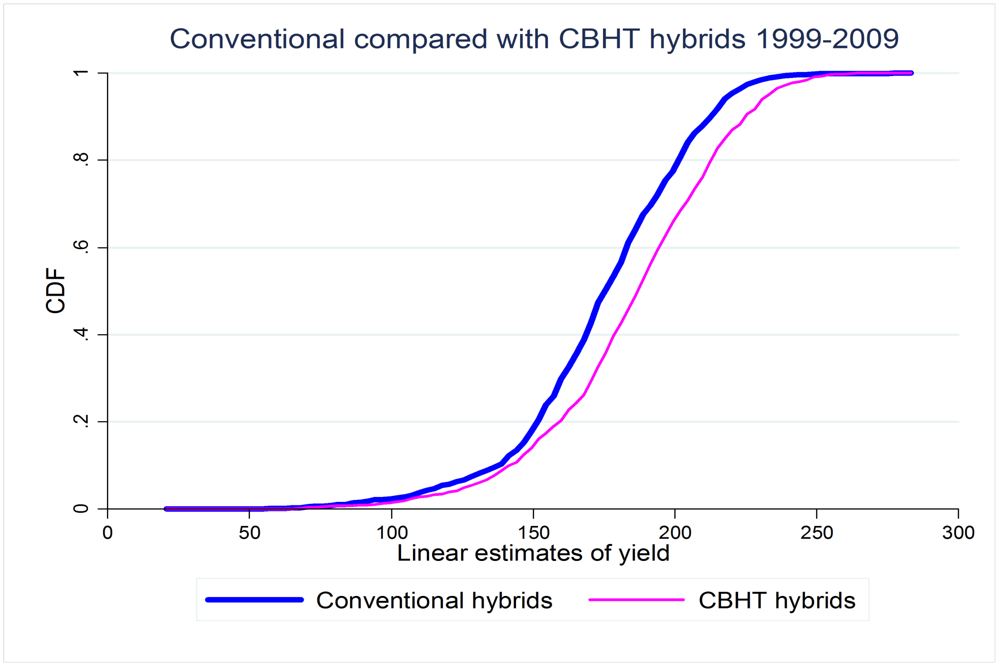

Fig B. Stochastic dominance: CBHT vs. conventional hybrids, 1999-2009

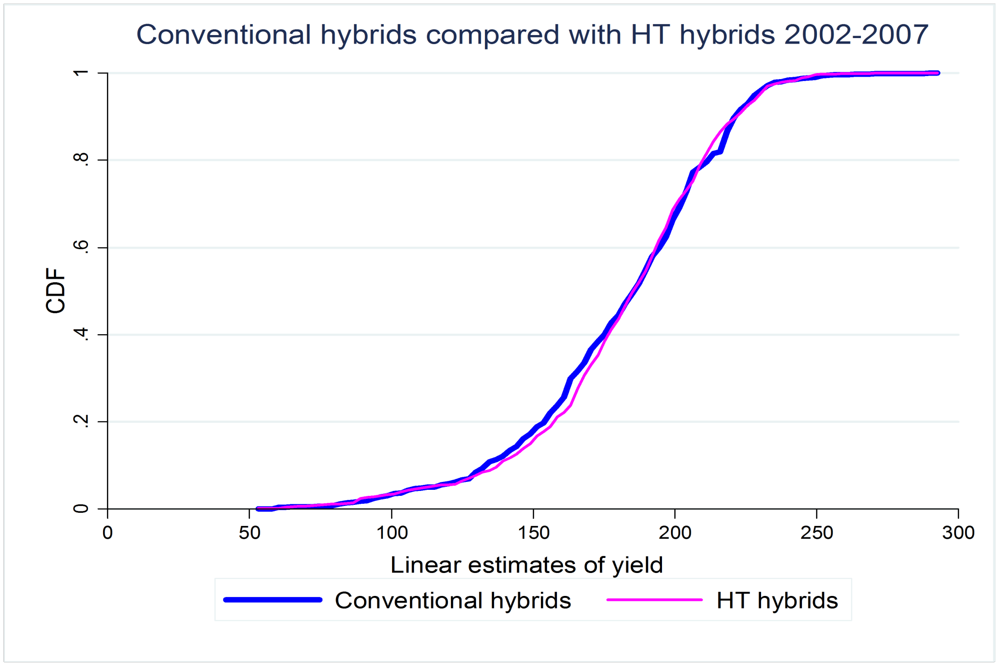

Fig C. Stochastic dominance: HT vs. conventional hybrids, 2002-2007

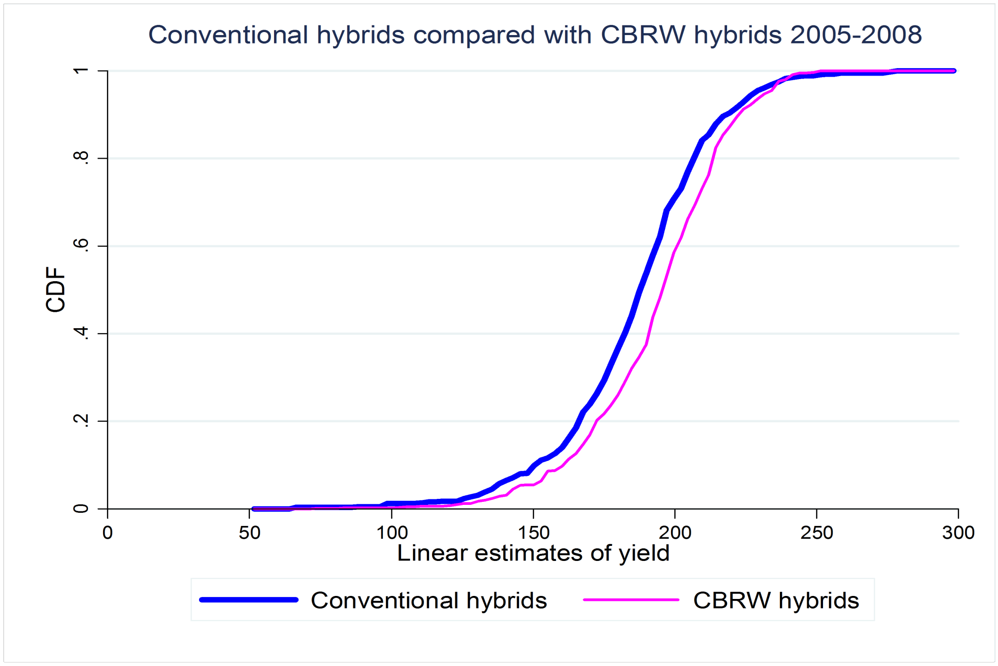

Fig D. Stochastic dominance: CBRW vs. conventional hybrids, 2005-2008

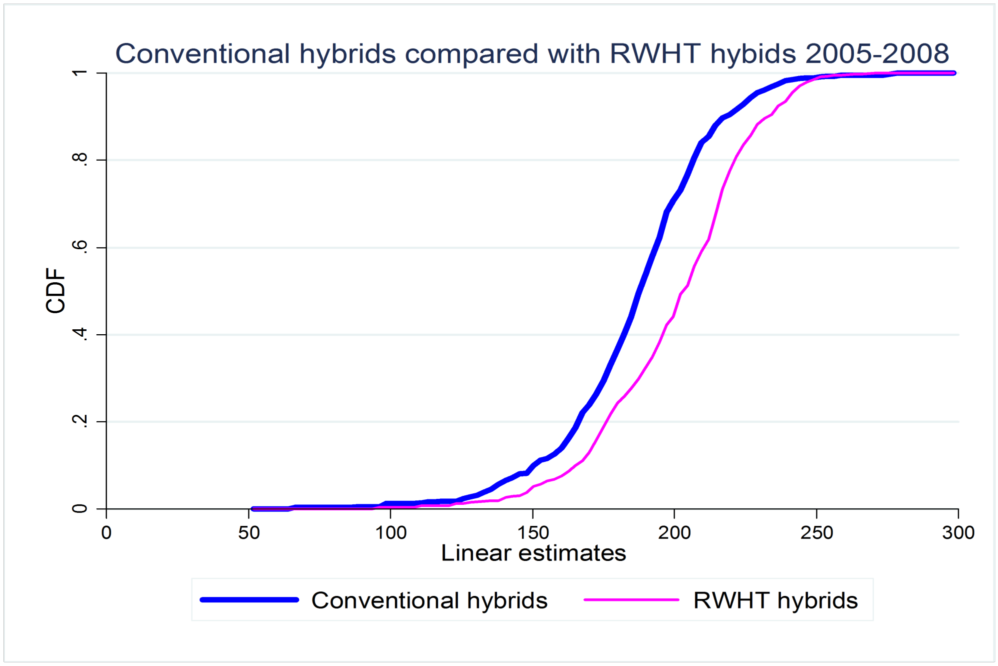

Fig E. Stochastic dominance: RWHT vs. conventional hybrids, 2005-2008
